# Thirty Years of Human Rights Study in the Web of Science Database (1990–2020)

**DOI:** 10.3390/ijerph18042131

**Published:** 2021-02-22

**Authors:** Priscilla Paola Severo, Leonardo B. Furstenau, Michele Kremer Sott, Danielli Cossul, Mariluza Sott Bender, Nicola Luigi Bragazzi

**Affiliations:** 1 Graduate Program in Law, University of Santa Cruz do Sul, Santa Cruz do Sul 96816-501, Brazil; priscillasevero@hotmail.com; 2 Graduate Program of Industrial Systems and Processes, University of Santa Cruz do Sul, Santa Cruz do Sul 96816-501, Brazil; sott.mk@gmail.com; 3 Department of Psychology, University of Santa Cruz do Sul, Santa Cruz do Sul 96816-501, Brazil; daniellicossul@mx2.unisc.br; 4 Multiprofessional Residency Program in Urgency and Emergency, Santa Cruz Hospital, Santa Cruz do Sul 96810-072, Brazil; maribendersott@hotmail.com; 5 Laboratory for Industrial and Applied Mathematics (LIAM), Department of Mathematics and Statistics, York University, Toronto, ON M3J 1P3, Canada

**Keywords:** human rights, co-word analysis, strategic intelligence, bibliometric analysis, SciMAT

## Abstract

The study of human rights (HR) is vital in order to enhance the development of human beings, but this field of study still needs to be better depicted and understood because violations of its core principles still frequently occur worldwide. In this study, our goal was to perform a bibliometric performance and network analysis (BPNA) to investigate the strategic themes, thematic evolution structure, and trends of HR found in the Web of Science (WoS) database from 1990 to June 2020. To do this, we included 25,542 articles in the SciMAT software for bibliometric analysis. The strategic diagram produced shows 23 themes, 12 of which are motor themes, the most important of which are discussed in this article. The thematic evolution structure presented the 21 most relevant themes of the 2011–2020 period. Our findings show that HR research is directly related to health issues, such as mental health, HIV, and reproductive health. We believe that the presented results and HR panorama presented have the potential to be used as a basis on which researchers in future works may enhance their decision making related to this field of study.

## 1. Introduction

The human rights (HR) concept embraces the essential rights of people considering only their condition of being human [1]. However, violations of HR still occur even after 70 years of the implementation of the Universal Declaration of HR. For instance, France and Denmark have been condemned for their restrictions of the clothing worn by some Muslim women in public spaces [2,3]; in addition, the Government of Azerbaijan has arrested civilians who have criticized the government [2]; furthermore, families have been separated at the borders of the United States [2,4]. The universal framing of human rights increases the amount of research in different areas of knowledge, such as health [5,6,7], children’s rights [8], domestic violence [9,10,11], and refugees [12].

These contexts require research capable of understanding the full dimensions of HR. In order to do so, the field of HR must be developed much more by researchers. Seven reviews were made with a general view of HR that identified the connection of the concept with human development [13] and barriers to its realization, including authoritarianism and social dominance orientation [14] and protection of the privacy of the violated [15], as well as perspectives to focus on people directly affected [16], on ways to limit threats between groups and counter the avoidance of conversations of rights violations [2], as well as forms to end the queer oppression [17].

Although the abovementioned studies provided relevant perspectives of HR for science, different approaches are still needed in order to advance the field, including bibliometric analysis. This approach can be used to enhance the understanding of HR paradigms; however, just one piece of research was found in the literature regarding HR which focused only on the health field [5]. In fact, no study in the health field of HR was found by performing a complete bibliometric review of HR using advanced techniques. The large amount of research and the time span across which the current publications are spread (1990–2020) indicates that a full bibliometric analysis is a suitable alternative to identifying the general panorama of the research over the years, which will help researchers to produce an overview of the field of research and identify its trends, as well as inform and support other researchers’ decision making. Therefore, our goal in this research was to answer three main questions:

RQ1: What are the strategic themes of human rights study
RQ2: What has the thematic evolution structure of human rights been?
RQ3: What are the trends and opportunities in the human rights field for academics and practitioners for future further study?

## 2. Methodology and Dataset

Bibliometric analysis is a technique used worldwide by researchers to better understand broad fields of study from the identification of important topics, evolution of themes over time, and trends, challenges, and opportunities for the development of future research. This analysis also makes it possible to analyze the performance of a field of study in terms of scientific productivity (e.g., authors, countries, universities). This information helps in decision making both for the development of new theoretical and applied works and for the generation of knowledge, which can be used to support new research [18,19,20,21,22,23,24,25,26,27].

### 2.1. Methodology

**Figure 1:**
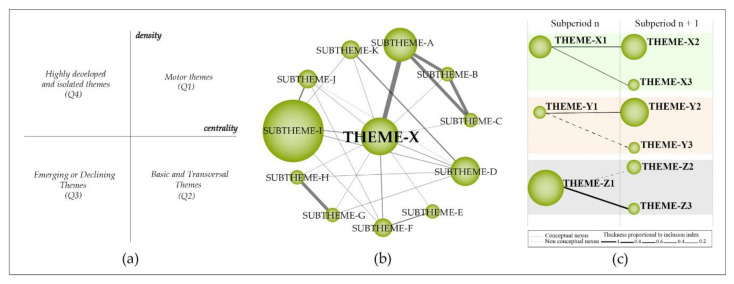
Strategic diagram (**a**); thematic network structure (**b**); thematic evolution structure (**c**).

In this survey, we conducted a bibliometric performance and network analysis (BPNA) into the field of research of HR by using the Science Mapping Analysis software tool (SciMAT) developed by the Department of Computer Science and Artificial Intelligence, CITIC-UGR (Research Center on Information and Communications Technology), University of Granada, Granada, Spain). We chose this tool due to the possibility to perform a complete science mapping analysis (from preprocessing to bibliometric network analysis), which presents a robust preprocessing step when compared with other bibliometric software (e.g., VOSviewer, Sci2tool). The BPNA method merges the techniques of performance analysis and science mapping. This union makes it possible to perform a broad scientific exploration into any research area using documents from scientific databases (e.g., Scopus, Web of Science (WoS)) and provide, as a result, an extensive overview and brand-new knowledge perspectives which can be used to aid decision-makers from universities and companies. We followed four steps to perform this research:

1. Detection of research themes: The research themes were identified using a frequency and network reduction of words. The clustering algorithm used was the simple centers algorithm. To normalize the data, Salton’s Cosine was used to create a strategic diagram and the equivalence index was applied to normalize the co-word network of the thematic evolution structure.
2. Depicting research themes and thematic network structure: The research themes were plotted in a bi-dimensional diagram (Figure 1a) composed of four quadrants, in which the “vertical-axis” characterizes density (D.) and the horizontal-axis characterizes the centrality (C.) of the theme. The research themes were classified into four groups: (a) motor themes (1° quadrant—Q1): high centrality and density; (b) basic and transversal themes (2° quadrant—Q2): high centrality and low development; (c) emerging or declining themes (3° quadrant—Q3): low centrality and density; (d) highly developed and isolated themes (4° quadrant—Q4): low centrality and high density.
3. Detection of thematic areas: The thematic network structure (Figure 1b) characterizes the co-occurrence between the research themes and highlights the amount of relationships (C.) and internal strength among them (D.). The thematic evolution structure (Figure 1c), provides an appropriate image of how the themes preserve a conceptual nexus throughout the following subperiods. The size of the clusters is proportional to the number of core documents; the links indicate co-occurrence among the clusters. Solid lines indicate that clusters share the main theme, and dashed lines represent the shared cluster elements that are not the name of the themes. The thickness of the lines is proportional to the inclusion index, which indicates that the themes have elements in common.
4. Performance analysis: The level of scientific contribution was measured by analyzing the important research themes and thematic areas using h-index, sum of citations, core documents, centrality, density, and nexus, among other themes. In addition, we conducted a performance bibliometric analysis of the field to identify the most productive researchers, institutions and journals.

### 2.2. Dataset

First, we defined the search string “human rights” to explore the documents present in the database. After this, we chose the (WoS) database, because it frequently indexes journals of high impact factor, as compared to Scopus, Google Scholar, and others. In addition, we chose to analyze only articles and reviews in the English language; therefore, a filter was applied. A total of 25,544 articles were found and exported from the WoS and imported to SciMAT. Then, we performed data preprocessing to exclude unwarranted information, such as duplicate documents (2) using Endnote, authors, journals, etc., using the SciMAT preprocessing tool. Along with the articles, 36,036 keywords were extracted and those with the same significance were grouped (e.g., “women’s health” and “womens health” (sic)); in addition, broad words, such as “law” and “human rights”, were excluded (760) because we wanted to find less well-known information. In this sense, 25,542 documents and 35,256 keywords were included for bibliometric network analysis. In addition, a preprocessing step was also applied to correct and group authors’ names, years of publication, and journals. Although the first article published and indexed in the WoS was done so in 1956, we identified that the documents present in the WoS were not keyword indexed until 1990. Therefore, for the analysis of the strategic themes, we considered only one period (1990–June 2020), and for the thematic evolution structure, we considered three different subperiods, namely, 1990–2000, 2001–2010, and 2011–June 2020.

Concerning the software parameters, we considered the authors’ words, sources’ words, and added words. In addition, a data reduction and a network reduction process were applied in order to consider the most important themes and exclude inappropriate keywords and co-occurrences. For the network extraction, we wanted to identify any co-occurrence in keywords. For the normalization, we used Salton’s Cosine for the strategic diagram and the equivalence index for the thematic evolution structure. A simple center algorithm was used for the mapping process. Finally, a core mapper was used, as well as h-index and sum citations.

## 3. Bibliometric Performance Analysis of Human Rights

In this section, we measured the performance of the field of HR in terms of publications and citations over time, including the most productive and cited researchers and the productivity of scientific journals, and countries, as well as the most important research areas found in the WoS. To do this, we used the following indicators: number of publications, sum of citations by year, journal impact factor (JIF), sand research field. For this, we examined the complete period under review (1990 to June 2020).

### 3.1. Publications and Citations Over Time

**Figure 2:**
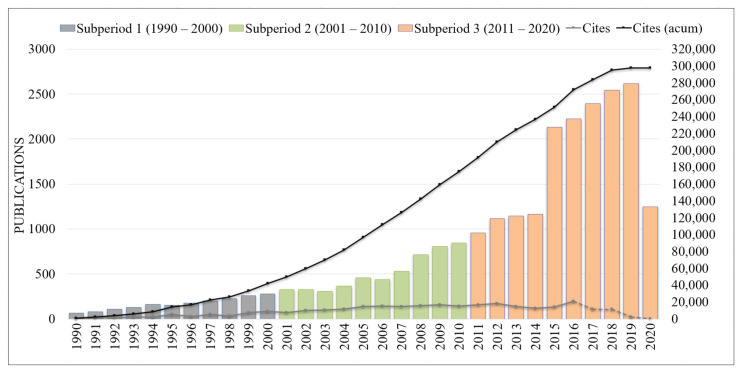
Number of publications on the topic of human rights over time (1990–June 2020).

Figure 2 shows the performance analysis of publications and citations in the HR field over time from 1990 to June 2020 in the WoS. The first subperiod (1990–2000) shows the beginning of the research field, with 66 documents and a total of 861 citations. This subperiod presents a slight increase until 2000. This slightly increase continues from the first subperiod until to the second subsperiod (2001–2010), with a total of 5121 publications and the highest number of citations, with 132,392 citations.

From the second to the third subperiod, it is possible to observe an increasing number of publications, rising to 17,546 publications, and a decrease in the number of citations, decreasing to 123,127 citations, that may happen because it can take three to seven years for a piece of scientific research to reach its peak point of citation [28,29]. The year with the highest number of citations was 2016.

### 3.2. Most Productive and Cited Authors

Table 1 displays the most productive and cited authors from 1990 to June 2020 in the HR field found in the WoS. Dr. Chris Beyrer has been the most productive researcher in the field of HR over this period, with 46 publications, followed by Sofia Gruskin (39), Stefan Baral (38), and Amanda Murdie (34). On the other hand, according to the WoS, Dr. Chris Beyrer is the only one of these who appears as both one of the top ten most productive and top ten most cited authors.

### 3.3. Productivity of Scientific Journals, Universities, Countries, and Most Important Research Fields

Table 2 shows journals that publish studies related to HR. *HR Quarterly* is the first ranked, with 731 publications, followed by the *International Journal of HR*, with 300, and the *Journal of HR*, with 277. On the other hand, the *Journal of Business Ethics* is the journal that has the highest journal impact factor (JIF) of those found, regarding 2019.

Table 3 shows the most productive institutions. The first ranked is the University of London, with 991 documents, followed by the University of California System, with 640 documents, and Harvard University, with 456 documents.

The analysis of the three tables shows that only Johns Hopkins University aggregates some of the most productive and cited authors, such as Dr. Chris Breyer and Stefan Baral at the Department of Epidemiology, and Lori Heise, at the Department of Population, Family and Reproductive Health. The institutions of the other authors were not found among those who most publish studies on the topic of human rights. It was also noted that the authors mentioned above have mostly not published their works in the journals that publish the most content on human rights (Table 2), according to the Web of Science database. The same occurs when we check the journals that the three most productive authors, Dr. Chris Beyrer, Sofia Gruskin and Stefan Baral, publish in (Table 1). In fact, for Dr. Chris Beyrer and Stefan Baral, the only journal found of those 10 most productive that is in their publications is *Health and Human Rights*; meanwhile, Sofia Gruskin, has published in *Health and Human Rights* and *Human Rights Quarterly*.

## 4. Science Mapping of Human Rights

**Figure 3:**
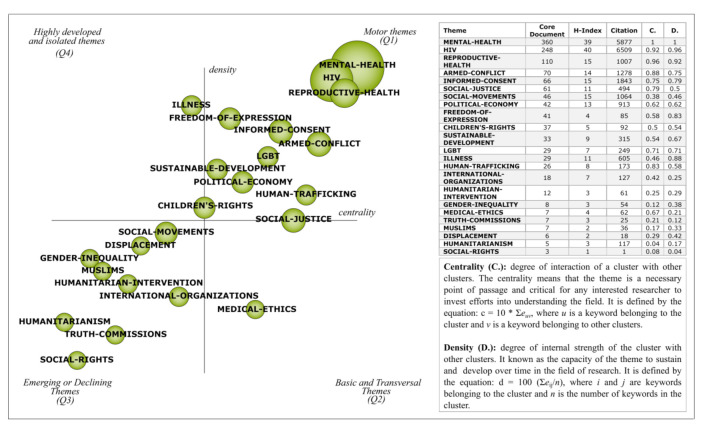
Strategic diagram depicting the performance of human rights.

Composed of four quadrants, the strategic illustration (Figure 3) presents 23 clusters, 12 of which are motor themes, 9 emerging or declining themes, 1 basic and transversal, and only 1 on highly developed and isolated themes. The size of the clusters represents the number of associated documents. Core documents, h-index, citations, centrality (C) and density (D) are presented for each cluster. The “mental health” cluster contains the largest number of associated documents (360) and has reverse combination of density and centrality that “reproductive health”, which is the first ranked in terms of centrality. Although “HIV” is not ranked the highest of the core documents, this cluster stands out as the most cited topic in the period (6509) and is ranked first in terms of h-index and density.

A broad view allows us to see that the motor themes are related to health and HR. In contrast, the emerging or declining themes discuss HR as a motor through which society can be developed, such as the discussions about “social movements”, “humanitarian intervention”, “gender inequality” and “social rights”. The main themes that guide the discussions in the HR field are discussed in depth in the following section.

### 4.1. Strategic Themes and Thematic Network Structure of Human Rights

#### 4.1.1. Mental Health

The “mental health” cluster approaches complex, distinct, and interconnected themes in different contexts and perspectives, including women’s rights [30,31], people with disabilities [32], human trafficking victims [33], domestic violence [34,35] and sexual violence [36,37,38]. In addition to these, traumatic events [39,40], “post-traumatic stress disorder” [41], “depression” [42,43] and “suicide” [44], are presented as the impacts that human rights violations can cause to mental health, which directly affect people’s “quality of life”.

This cluster also shows a co-occurrence with the subtheme of “refugees” which also connects to both the “asylum seekers” and “immigration detention” subthemes, and particularly affects countries such as South Africa [45], Israel [46], Iraq [47], and Syria [48]. In this sense, thousands of people are forced to leave their country to pursue a new life in order to save their physical and mental integrity, considering the circumstances of violence, discrimination, hunger, and living conflicts in which they find themselves at home.

Body narratives can be understood from the intersection of issues involving mental health, migration, and HR [49]. This is justified by the aspects that affect the wellbeing of refugees or asylum seekers, as they experience a displacement processes, the loss of the feeling of belonging to a geographical space, the distance from their family and cultural environment, and their exposure to situations of violence and xenophobia [50,51]. There are specific risks and exposures which often occur during the migration trajectory [52] which cause a prevalence of high levels of distress, life dissatisfaction [51], anxiety, depression, and suicidal ideation [53,54]. It is possible to infer that the subjective aspects of the psychological suffering of refugees are intrinsically related to the feelings of exclusion and the methods of oppression and violence they face.

In addition, this cluster also shows that mental health can also be affected by the strong relationship between the subthemes “domestic violence” and “intimate partner violence”, since these violent factors are considered a major health problem that induces physical and psychological illnesses on their victim as a consequence [55]. Such situations also represent human rights violations against their victims in their mental, physical, sexual, and reproductive health [56].

#### 4.1.2. HIV

The “HIV” cluster shows connections among the subthemes “sex work”, “sexually transmitted infections”, “risk behavior”, and “HIV-prevention”. The main behaviors associated with human immunodeficiency virus (HIV) infection are the practice of transactional sex [57,58], use of injectable drugs [59], absence of condoms, and sexual violence [60]. In addition, vulnerabilities such as poverty, discrimination against sexual minorities, mental health problems, incarceration, and detention are connected to HIV incidence [61,62,63]. Furthermore, countries that discriminate against sex professionalization reflect a lack of safe care in terms of confidentiality, preventing access to public resources for HIV treatment [64].

In fact, the number of HIV infections has increased by more than 60% in 50 countries worldwide, since 2010 [65]. The strong co-occurrence between the “condom use” and “sexually transmitted infections” subthemes is also a concern, due to the low trend of condom use among young people [66,67,68]. In order to help resolve this, it is necessary to increase the level of sexual education in place lacking in this regard, and increase the awareness regarding one’s personal perception of the risks of this infection as a method of HIV prevention [65,66].

In addition, the co-occurrence of the great number of documents associated with the subtheme “public health” reveals the need to reaffirm that health is a fundamental right. In fact, the vitality of legal protection for sex workers and social minorities is considered to be a protective and empowering way to overcome discrimination [69]. In this sense, community meetings that foster respect and support [70], education regarding sexually transmitted diseases, access to HIV prevention methods, gender assertion care, and stigma reduction [61] are considered essential for this neglected population. In terms of control, the application of a universal HIV test expressly promotes the importance of health spaces [70].

#### 4.1.3. Reproductive Health

The “reproductive health” cluster encompasses diverse and cross-cutting themes. In this way, reproductive health is connected to “gender equality”, considering women’s position in society often being seen as inferior, based on chauvinist cultures. This context reinforces the “sexual violence” cluster and the victim blame culture, alongside difficulties in differentiating consensual sex from forced sex, in addition to unplanned pregnancies that create feelings of guilt and shame, which also generate stigma in society and a step to depression and anxiety causes [64].

From this perspective, women’s “reproductive rights” are stunted, considering that, in many countries, unplanned pregnancies force them into forced marriages, even in adolescence [71]. As a consequence, discussions about “abortion” legalization have increased, as this is considered an alternative to the failure of “contraception” methods or the lack of access to them. However, in order for this to be successful, both access to contraceptives and safe abortion are important to prevent maternal deaths and also represent society’s ability to respect women’s decisions and guarantee their health and rights [72].

In general, “sexual health” and “women’s health” are harmed by cultural biases, such as cases of “female genital mutilation”, followed by tradition; this leaves women susceptible to recurrent infections of the urinary tract, dysmenorrhea, sexual problems, infertility, and childbirth complications [73]. Therefore, there is an urgent need for discussions that consider the strong relationship of HR in health and the barriers to its access [74].

#### 4.1.4. Armed Conflict

Armed conflicts are a part of global history, with 182 wars, in addition to minor conflicts, recorded up until the year 2017 [75]. In addition to the deaths caused by wars, “military” conflict can induce refugee flows, migration, capital flight, and also the destruction of a societies’ infrastructure [76]. In order to combat these issues, the laws of “international humanitarian law” become applicable during an international or non-international armed conflict, according to General Comment on Article 4 of the International Covenant on Civil and Political Rights.

Moreover, the connection between “political violence” and “rape” represents one of the “women’s rights” violations that prevails in armed conflicts [36], due to “gender-based violence”. In fact, in some armed regions of conflict (e.g.., the Democratic Republic of the Congo (DRC)), women and young girls have been kidnapped, raped, and become trapped as sex slaves [77]. In addition to that, there have been reports of men being forced to rape women of their own family [77]. This kind of violence against women is forbidden under international humanitarian law [36]: Rule 93 of this document states that rape and other forms of sexual violence are prohibited [78]. In addition to that, studies of environments of armed conflict also indicates that women are more predisposed than men to feel distress and develop mental illness in such situations, including post-traumatic stress disorder (PTSD) [79,80,81,82,83].

Governments are striving to seek alternative forms of “conflict resolution” in order to reduce the violence of armed conflicts through the adoption of new techniques and societal procedures [84]. The presence of democratic dyads can reduce the levels of armed conflict and also represents the most efficient method of conflict resolution [85]. In addition, over the last few years, the practice of mediation has been supported as a technique to resolve armed conflicts through international organizations [86]; however, this mechanism needs further development and an increase in its scope of involvement [86].

#### 4.1.5. Informed Consent

“Informed consent” is a term relating to people’s autonomy, allowing them to live life as their own protagonists, as well as being able to define their own participation—or lack of participation—in projects and programs, in addition to their ability to perform procedures that relate to their own bodies. In this sense, this cluster encompasses the human right of guaranteed access to health care, from a “bioethics” perspective. In addition, the abstract character of fundamental rights allows judges or legislators to make different decisions in different cultural contexts, according to each situation presented, such as in cases of abortion, assisted suicide, or genetic improvement [87].

“Human dignity” violations were the basis for the development of HR [87], and these concepts are understood through social, political, legal, and moral viewpoints, considering that the “development and progress of society and culture is impossible without full-fledged human personality with a sense of self-worth, dignity, freedom and rights” [88].

Furthermore, researchers are putting their efforts towards discussing and defining the principles of human dignity and the guaranteeing of human rights of vulnerable groups, based on the difficulties that these groups could have in discerning procedures or treatments and giving their consent (e.g., “indigenous peoples”), or people who are currently in a vulnerable situation (e.g., “pregnant women”). The cluster also shows a co-occurrence among the “impact assessment”, “indigenous peoples”, and “indigenous rights”, revealing further concerns about the human right of those people belonging to these groups. The right to free prior informed consent (FPIC) is protected by the United Nations (UN) Declaration on the Rights of Indigenous Peoples and the international HR agenda for indigenous people [89].

This class of rights has seen violations caused by paternalism for a long time; therefore, it is important to discuss ways to guarantee these fundamental rights [90]. In this context, there has also been discussions among researchers regarding the guaranteeing of human dignity to elderly people [91], the non-voluntary hospitalization of psychiatric patients [92], and tourism projects in villages without the consent of the indigenous people who live there [93], including the aborigines [94].

#### 4.1.6. Social Justice

**Figure 4:**
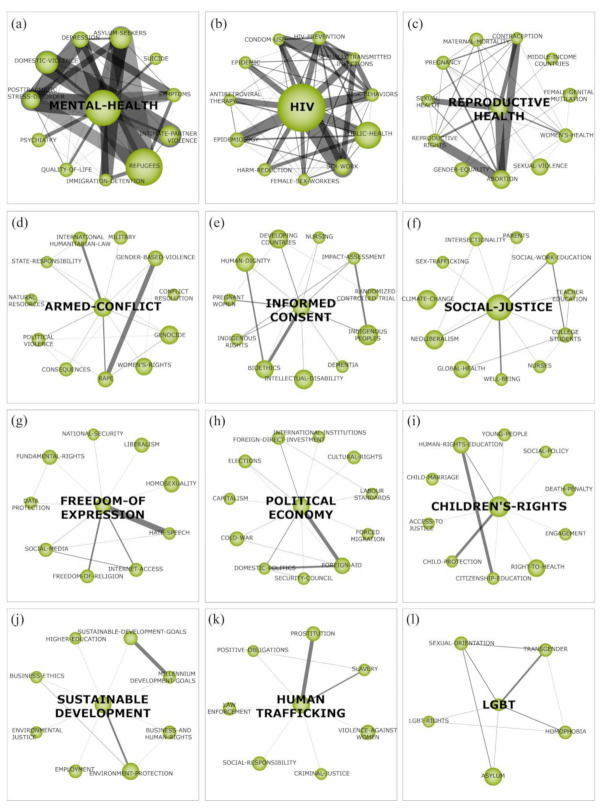
Strategic diagram depicting the performance of human rights research. (**a**) the cluster ‘mental-health’. (**b**) the cluster ‘HIV’. (**c**) the cluster ‘reproductive health. (**d**) the cluster ‘armed-conflict’. (**e**) the cluster ‘informed consent. (**f**) the cluster ‘social-justice’ (**g**) the cluster ‘freedom of expression’. (**h**) the cluster ‘political economy’. (**i**) the cluster ‘children’s rights’ (**j**) the cluster ‘sustainable development’. (**k**) the cluster ‘human trafficking’. (**l**) the cluster ‘LGBT’.

Social justice aims to ensure fair relations in society, the enforcement of civil rights, and appropriate individual and collective conduct, and is directly related to the promotion of HR [95]. Social justice faces several structural factors and challenges that make HR available to only a small part of the world population [96]. This reinforces the importance of social workers and processes to integrate marginalized peoples into society and promote equal rights, both economically and socially [97]. In this sense, social development is an excellent way to deal with societal challenges and injustices and to promote social justice and wellbeing [96]. Through thematic network structures, we identified that “social justice” (Figure 4f) has a strong relationship with education issues (e.g., “college students”, “teacher education”, and “social work education”). The relationship between these clusters occurs because education supports ethical and moral construction in children and young people, and consequently, the social development of communities and societies [98]. The “parents” cluster appears to be related to the presence of parents in their children’s education [99], and in the relationship between parents and children, society’s expectations and the importance of parenthood and obligations towards society are found [100].

Social justice also presents links between the themes of “sex trafficking” and “WELLBEING”, and there is also an association between these and clusters related to healthcare (e.g., “nurses” and “global health”), which includes studies related to public health provision for all individuals [101], social inclusion, and mental health services [102,103,104,105]. In addition, “intersectionality” is discussed as a means by which to assist in integration theory and practice from different areas of social justice [106,107,108]. Other concerns are raised through the “neoliberalism” cluster, which discusses resistance to neoliberalism in social work education [109], and the “climate change” theme that represents the concern with the environmental pillar of sustainable development for the wellbeing of the population and the future of humanity.

#### 4.1.7. Freedom of Expression

“Freedom of expression” has information as its main axis, which guides citizens and impacts on education, employment, and social relations, and for this reason, it is considered a necessary human right [110]; it is also the base of democracy [111]. On the other hand, there is a limit to freedom of expression, “hate speech”, and the incitement of violence, all of which represent offensive and harmful forms of communication [112], and are often interrogated, mainly by legal entities; however, these legal entities also need to avoid censorship at the same time as stopping these forms of speech that harm or put individuals or groups at risk or provoke situations of violence. The strong co-occurrence between freedom of expression and hate speech also represents that, while discourse can be considered as freedom of speech and an individual right, hate speech is considered to be a variant of these which constitutes a violation of fundamental rights [112]. The impacts of hate speech and the control of such hate speech can be different in different situations, and can be measured based on the context of the person, location, and circumstance, among other factors [112].

In addition, “social media” has an important role in the context of hate speech, as it has the ability to spread information at great speed and reach a large number of people worldwide. For many years, social media platforms were considered to be democratic, as they facilitate freedom of expression and access to varied information; however, it has also contributed to the rapid spread of hostility, hate speech, discrimination, and illegal content [113], mainly content featuring religious intolerance and intolerance against homosexuals [114]. In order to counteract this “fundamental right”, the access of users’ rights to protection from discrimination must be increased, in addition to the required increase of the transparency and responsibility of social media companies, in order to expand the democratic values present in these internet environments [111].

#### 4.1.8. Political Economy

The political economy is concerned with the production and commercialization of products and relationships between these factors and laws, as well as political, cultural, demographic, and economic variables [115]. The “political economy” cluster (Figure 4h) is related to political regimes (e.g., “capitalism”) through works which discuss corporate responsibility and the value of work under the capitalist system [116]. Researchers present opposing opinions on the impact of capitalism on HR: on the one hand, capitalism is seen by some as destructive for society; on the other hand, it is discussed by others as a plausible alternative for human development through organizations [115]. These different opinions of researchers reinforce the need for in-depth case studies in organizations and across society to understand the effectiveness or inefficiency of the capitalist system regarding the development of HR [115]. In addition, the “Cold War” cluster contains studies that analyze the transformations seen in HR since the Cold War [115,117].

Other clusters include issues related to political factors (e.g., “elections”, “security council”), and discuss political regimes and governance [118], and the impacts of national and international policies on culture, migration, organizations, and workers (e.g., “domestic politics”, “forced migration”, “cultural rights”, “foreign direct investment”, “foreign aid”, “international institutions”, “labor standards”). These clusters present studies on organizations’ codes of ethics, including socioeconomic, legal, and political factors [119], in addition to approaches and regulations for transnational farmland acquisition [120]. Kotikalapudi [121] presents research on the political economy of corruption and crony capitalism in the Bangladesh coal sector, and the impact these factors have on the country’s democracy; in addition, DeMeritt and Young [122] explore the political economy of HR in the oil and natural gas sector. Political and economic determinants have also been discussed in regard to foreign direct investment and its relationship to workers’ skills, market size, and political instability [123]. In addition, the rights of migrant workers and the transnationalization of social relations are explored by Elias [124], in order to understand the importance of social relations in the labor market, perspectives on labor rights, and the rights of migrant women.

#### 4.1.9. Children’s Rights

Children are developing human beings, and because of this condition of vulnerability, require special protection, which may come from parents, family, and the communities in which they live [125], as seen in the “child protection” cluster. In this sense, societal and HR development have made it possible for children, who were previously not considered holders of human rights in the same way as adults, to become full citizens and holders of HR, including the rights to expression and participation in decisions regarding their parents [126]. This relationship is based on the strong connection between the clusters of “human rights-education” and “citizenship education”, as well as being related to the “access to justice” subtheme. In this sense, education is a fundamental right of the child, but also a valuable vehicle with which all the other HR may be achieved [127].

In order to achieve these goals, the 1948 Universal Declaration of Human Rights demonstrates an advance in social rights legislation and also represents an international document that features a strong protection of HR as its basic content. Its text provides the foundations for the formation of the primary protection of childhood in Article 25 [128]. In addition, the UN Convention on the Rights of the Child, among others, also states that children are entitled to special care and assistance in their lives [129].

Despite these factors, the “children’s rights” cluster (Figure 4i) shows that research on HR violations of children related to “child marriage” are still widely discussed by researchers [130,131,132], showing that child marriage still occurs, despite that it is a recognized health and HR violation [133]. The poorest and least educated girls are those most affected and vulnerable to child marriage [133]. According to UNICEF research, there are 650 million girls alive who were married as children and 12 million under-18-year-old girls are married each year [134]. In response, support for the health, particularly the reproductive health, of young girls must be provided in parallel with efforts that seek to reduce and eliminate the child marriage [133]. Therefore, the protection of children’s rights requires “social policy” capable of promoting their full and healthy development [135].

#### 4.1.10. Sustainable Development

In 1987, the Brundtland Commission Report defined sustainable development as a way to meet the needs of the present society, without harming future generations [136,137]. At the beginning, the concept of sustainable development represented efforts to integrate economic and environmental development [136], and the social dimension was only added in 1990 through the Human Development Reports [138]. The challenges related to environmental degradation, climate change, urbanization and socio-economic inequality increase the need to incorporate paradigms such as sustainable development to create ways to deal with the inherent environmental risks, social instability and resource depletion [139]. In this sense, the cluster ‘SUSTAINABLE-DEVELOPMENT’ (Figure 4j) is a motor theme concerned with social, economic, and environmental development, and has strong association with clusters related to challenges and goals of the sustainability (e.g., ‘MILLENNIUM-DEVELOPMENT-GOALS’, ‘SUSTAINABLE-DEVELOPMENT-GOALS’, ‘ENVIRONMENT-PROTECTION’, ‘ENVIRONMENTAL-JUSTICE’ and ‘HIGHER-EDUCATION’). Global targets of the Sustainable Development Goals (SDGs) and the Millennium Development Goals (MDGs) are part of the 2030 Agenda for sustainable development for economic growth, human well-being, and environmental protection [140]. The 2030 Agenda represents an inclusive action by organizations, governments, and society for the sustainability of the planet, seeking to ensure the human dignity, equity, and equality [141].

In addition, the concern of sustainable development with HR includes the legal rights to education, mainly with a focus on equitable and inclusive quality education for vulnerable groups and ethnic minorities, elimination of gender disparities, greater cultural diversity, and employment [142]. The relationship of sustainable development with ‘BUSINESS-AND-HUMAN-RIGHTS’, ‘BUSINESS-ETHICS’ and ‘EMPLOYMENT’ show the corporate social responsibility related to economic, societal, and environmental sustainability. For this, organizations seek cooperation, social relationships, ethical behavior [143], sustainable resource management and mechanisms for environmental protection [144]. Issues such as the relationship between employee and employer, employee involvement, decent work, laws and employer’s rights are widely discussed [145,146]. In this sense, this cluster represents the integration of the sustainable development tripod, encompassing the three Ps of Profit, Planet and People [138].

#### 4.1.11. Human Trafficking

Human trafficking, also known as “modern slavery” [147], is a form of transnational crime that is one of the most lucrative forms of organized crime [148]. The practice of human trafficking can be defined as forced, coercive, and fraudulent recruitment of a person or people for work and services, including sex work, that cannot be left at will [149,150,151] and that can cause multiple physical and psychological consequences for victims, such as depression, anxiety, and post-traumatic stress disorder [151,152,153,154].

According to International Labor Organization, the worldwide current estimate of human trafficking claims that 20.9 million people are currently subjected to forced labor, of which 11.4 million (55%) are women and girls, as compared to 9.5 million (45%) men and boys. The research also shows that 15.4 million (74%) of the total number of people human trafficked are adults, which indicates that adults are more affected than children. In addition, 90% the total victims are exploited by the private economy, 68% are forced to work in economic activities such as domestic activities and agriculture, 22% are forced into sexual exploitation, and 10% are forced into state-imposed forms of exploitation, for instance, in rebel armed forces.

The large number of women trafficked for forced sex work is represented by the strong connection between the cluster and subtheme “prostitution”, since sex trafficking is considered one of the most severe forms of human trafficking [155], and is also one of the fastest growing criminal branches [150], also being represented by the subtheme “violence against women”. Despite the global prohibitive norms regarding the prohibition of “slavery” (e.g., the Universal Declaration of Human Rights and International Covenant on Civil and Political Rights), the bigger link with this subtheme indicates that there is an increase related to slavery in its spread and geographical reach, which has been shaped and increased by globalization [156]. This is the reason why “positive obligations” of anti-human trafficking are necessary in order to prevent its occurrence [148,157].

#### 4.1.12. LGBT

The struggles to guarantee the rights and dignity of sexual minorities have repercussions worldwide. For instance, Canada has been considered the international vanguard of legal equality for sexual minorities since it signed the Canadian Charter of Rights and Freedoms and the Hate Crimes Law, and recognized gay marriage [158]. However, stigmatizing barriers have yet to be overcome; attitudes that favor those of cis gender prevail in countries including the United Kingdom, where trans people are forced to reveal their “gender history” before marriage [159]. In addition, the United States, Brazil, Mexico, and India all have health services considered unsafe, scarce, and inadequate for the potential needs of transsexuals [160,161].

This cluster also has a strong connection with the sub-theme “sexual orientation”, the scenario of which can show extremely discriminatory attitudes that argue that sexual diversity is a genetic error or a psychological “disturbance” [162] that defies acceptable behavioral patterns. In this sense, the oppression of sexual minorities is understood as a transgression of HR and permeates the life experiences of LGBT people, causing damage to their physical and mental health at significantly high levels as compared to the general population [160,163,164].

In addition, the co-occurrence of the subtheme “sexual orientation” with the subtheme “homophobia” shows that the countless attempts made to harm LGBT people continue today. For instance, research conducted in Latin America, North America, Europe, and Australia, focused on occurrences of homophobia in the context of schools, emphasizes that children and adolescents receive their school marks in the short, medium, and long terms through the incidence of anxiety, personality disorders, and chronic depression [163,165], all of which can be exacerbated by such discrimination as homophobia. This issue is also a concern in Jamaican society, regarded by some as among the most homophobic and transphobic societies worldwide, constituting an endemic condition, a public health problem associated with family rejection, juvenile detention, and sexual abuse [166,167].

### 4.2. Thematic Evolution Structure

**Figure 5:**
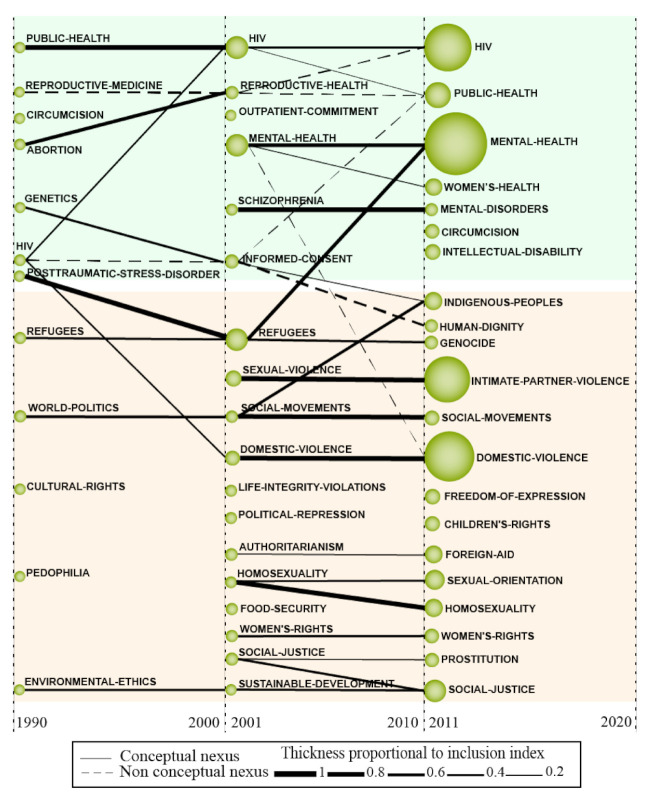
Thematic evolution structure of human rights study from 1990 to June 2020.

The analysis of the thematic evolution of themes inherent to HR research (Figure 5) contributes to the visualization of its predominantly evolved or emerging concepts, as well as their connections, over the years. Thematic evolution maps are read using the following information [168]: the solid lines refer to linked clusters that share a main item, the dashed lines refer to themes that share elements that are not the main item, the thickness of the edges is proportional to the inclusion index, and the size of the clusters is proportional to the number of related articles associated with each cluster.

This evolution map was divided into three subperiods, namely, 1990–2000, 2001–2010, and 2011–2020. In addition, it presents two thematic areas; the green field encompasses 20 clusters focused on aspects that contemplate the relationship between health and HR, four of which are characterized by individual performance; in other words, the themes do not have interrelationships. The yellow field covers 31 clusters that list HR, and of these, seven are presented in isolation.

#### 4.2.1. Health Related Themes of Human Rights

In the first subperiod examined, several cross-cutting themes are discussed, such as “public health”, especially with regard to global policies, which have a major impact on guaranteeing citizens’ rights. There were also studies found on issues related to the sexual life of individuals, (e.g., “reproductive medicine”, “abortion”, and “circumcision”), which is sometimes considered a taboo topic, considering the strong cultural and social influences, which also end up impacting on the fundamental rights of individuals. During this subperiod, several activities regarding “HIV” emerged, motivated by the inclusion of the disease caused by this virus in the International Classification of Diseases and by the increased use of antiretroviral drugs.

In the second subperiod examined, the relationship between “public health” and “HIV” is the most prominent, since these is characterized by their sharing of themes over the three subperiods. In addition, two of the themes with the most significant number of works are reminiscent of the first subperiod, such as “HIV”, the studies of which were stimulated by the exponential increase of contamination by the virus and the lack of a cure found, becoming a severe problem of “public health”; and “refugees”, which denotes the concerns regarding mass hunger and wars that compel people to flee their homes. Another relevant theme in this subperiod is the issues related to the broad field of “mental health”, the preservation of which is understood as a basic human right and must be guaranteed through public policies. Also relevant are the themes of “sexual violence” and “domestic violence”, which are intrinsically related to “mental health”, and mainly with the right to human dignity. In addition, studies relate “domestic violence” to “HIV”, which elucidates the need for public policies that address this issue.

In the third subperiod examined, “mental health” becomes the most relevant subtheme of all, featuring the largest number of studies in HR, which is explained by the fact that the theme is based on struggles aimed at guaranteeing rights, considering the often-seen attempts at annihilation, discrimination, and the violation of singular experiences as “abnormal”. In this sense, it is understood that everyone has the right to good mental health; at the same time, it is understood that people’s mental health suffers interference from violations of other human rights. This theme also has connections to “sexual violence”, “domestic violence”, “refugees”, and “post-traumatic stress disorder”, in which the reality of the people who have been violated in regard to their rights to housing and geographic belonging, stands out most of all, an aspect that contributes to the triggering of intense mental suffering. In addition, discussions about “HIV” that started in the 1980s continue to grow during this third subperiod, increasing their visibility, especially as a consequence of discussions about policies aimed at preventing contamination. The correlation between the “genetics”, “informed consent”, and “indigenous peoples” clusters express the problems experienced by those who have been affected by human rights violations, especially due to cultural differences.

#### 4.2.2. Themes of the Human Rights Field

In this article, we analyzed the evolution map in order to identify the interrelationships between the themes over time. Mapping this scientific evolution allows us to understand how the abovementioned subthemes have been studied and evolved over time, and provides a clear view of the themes that have developed and those that have declined in the field of study of HR [168]. To analyze the scientific evolution of the study of HR, we generated an evolutionary map (Figure 5) using the SciMAT software. The size of the clusters is proportional to the number of associated documents found, while the shape and thickness of the lines represents the link force between the themes from one period to another.

Regarding clusters of the general concepts of HR, we can observe that the clusters “cultural rights” and “pedophilia” disappear after the first subperiod (1990–2000). The other clusters in the first subperiod are maintained or transformed over time. The “refugees” cluster remains in the second subperiod (2001–2010) and evolves into concerns related to “mental health” in the third subperiod (2011–2020). The “world politics” cluster has a strong relationship with the “social movements” cluster that appears in the second and third subperiods and is linked to the “indigenous peoples” theme. The “environmental ethics” cluster is related to the “sustainable development” issues of the second subperiod.

It is interesting to note that, in the second subperiod, the number of clusters associated with HR increases exponentially and remains high in the third subperiod. This increase highlights the growing concern and relevance of issues related to HR over time. In the second subperiod, the discussions cover new themes, such as sexual and domestic violence, political issues, women’s rights, homophobia, and food insecurity. In the third subperiod, it can be observed that the themes that characterize the most recent concerns are related to HR; in this subperiod, the emerging themes appear as “freedom of expression” and “children’s rights”, while the themes already discussed in the previous subperiod appear to increase in the frequency in which they are mentioned, along with an increase in the volume of associated documents, such as “domestic violence”, and “homosexuality”, “social movements”, and the evolution of the theme “intimate partner violence”. The importance of these themes highlights issues of physical and moral violence that were once normalized in many societies and are currently being exposed and discussed in the directions of social justice and equality.

## 5. Conclusions

The goal of this research was to conduct a BPNA of HR research present in the Web of Science database. Our results show the main topics discussed by academics in the HR field. The strategic diagram presents the main themes discussed in terms of centrality and density, depicted in which are the motor themes (Q1), which were investigated in-depth via a review of the thematic network structures of each theme found. In addition, the thematic evolution structure presented the most developed clusters over time. Such results made it possible to understand how the field of research of HR is evolving over time, and it was possible to predict future trends by analyzing the associations of HR with healthcare concepts such as mental health, HIV, and reproductive health; therefore, we suggest that future works relate to these topics. In addition, we also recommend future works attempt to research other emergent themes (Q3) in order to avoid the mitigation of themes that are currently immature, such as social movements and gender inequality. The limitations of this work also must be highlighted: for instance, only the Web of Science database was used, which could serve as a limitation. In addition, only articles written in the English language were used; furthermore, only those papers which presented keywords indexed in the databases were used. Although SciMAT presents a complete mapping of the science, future works should be conducted that also use other bibliometric software packages, such as VOSviewer, Sci2tool and SiteSpace, in order to present different points of view, since we did not find any bibliometric analyses related to general HR. We hope that this research will form the basis for future research in the field of HR.

## Figures and Tables

**Table 1:** Most cited/productive authors from 1990 to June 2020.

Author Citation	Number of Citations	Author Productivity	Number of Publications
Garcia-Moreno, Claudia	2858	Beyrer, Chris	46
Heise, Lori	2781	Gruskin, Sofia	39
Ellsberg, Mary	2694	Baral, Stefan	38
Jansen, Henrica	2694	Murdie, Amanda	34
Watts, Charlotte	2694	Silove, Derrick	32
Beyrer, Chris	2313	Vanclay, Frank	25
Poe, SC	1717	Amon, Joseph J.	23
Thornicroft Graham	1261	Peksen, Dursun	21
Risse, T.	1192	Cole, Wade M.	20
Hafner-Burton, Emile	1078	Noorani, AG	20

**Table 2:** Journals that publish studies on the topic of human rights.

Journal	Doc.	JIF
*Human Rights Quarterly*	731	0.841
*International Journal of Human Rights*	300	-
*Journal of Human Rights*	277	1.185
*Health and Human Rights*	188	1.407
*American journal of International Law*	175	1.667
*Netherlands Quarterly of Human Rights*	168	-
*European Journal of International Law*	140	1.011
*Human Rights Law Review*	133	-
*Journal of Business Ethics*	127	4.141
*International Studies Quarterly*	127	2.172

**Table 3:** Institutions that publish studies on the topic of human rights.

Institution	Doc.
University of London	991
University of California System	640
Harvard University	456
University of Oxford	380
University of Toronto	355
Columbia University	321
University of New South Wales Sydney	251
University College London	230
University of Melbourne	229
Johns Hopkins University	226

## Data Availability

Not applicable.
